# Racial Proclivities to Disease

**Published:** 1909-10-30

**Authors:** 


### The Hospital

A Journal of

The Medical Sciences and Hospital Administration.

New Series. No. 140, Vol. VI. [No. 1212, Vol. XLVII.] SATURDAY, OCTOBER 30, 1909.

### Racial Proclivities to Disease.

An article recently appeared in a contemporary dealing with the incidence of tuberculosis among the Jewish population, and touching the problem of racial proclivities to disease. There is no doubt that tuberculosis in general takes a far lesser toll of our Jewish than of our Gentile population. During the decade 1891-1900 phthisis was responsible for 10 per cent. of the total deaths in St. George's-in-the-East and for nearly 11 per cent. in Bethnal Green, while in Stepney, where the bulk of the Jewish colony resides, only 6.4 per cent. of the deaths were due to this disease. In commenting upon these and similar figures, the writer of the article observes that racial immunity probably plays a part in producing the result, the Jews being notoriously refractory to bacterial diseases, but that other elements must contribute to their security. These elements he finds in their habits of life, habits dictated partly by the rules of their religion, and partly by the instinctive genius of the people.

It is clear that a race whose whole history has been one of oppression endured and surmounted must be possessed of some capacity to resist the lethal influences under the stress of which other oppressed races in the world's history have been eliminated, and it is a matter of ethnological importance to determine as far as possible to what extent this resistance is innate and to what extent due to wise rules of life which can be imitated. The racial immunity to bacterial disease which is claimed for the Jews undoubtedly exists. It is a frequent comment of those engaged in medical work among the Jews of the East End that the Jew will survive lesions produced by the pyogenic cocci under circumstances which would certainly have proved fatal to a Gentile. The statement is, of course, a mere opinion, but it is one widely held by persons of experience and must be accorded some weight. It is not, however, necessary to press this point, for the habits of life among the Jews supply many important factors tending towards racial survival. They marry young and their families are large—an essential item, allowing a good margin for casualties. But the casualities which decimate each generation of our own people during the years of infancy find no parallel in the history of a Jewish family, be it ever so poor. Nor is the reason far to seek. It is a rarity to meet a Jewish mother of the working-class who does not feed her baby at the breast, while among our own people of similar status, the practice is becoming daily less universal. As a consequence of this natural feeding, the Jewish infant escapes to a large extent, not only the immediate danger of death from zymotic enteritis, but also the dangers incidental to a constitution weakened by an exhausting, even though not fatal, disease.

When we come to consider the causes which give the Jewish mother this pre-eminence in infant-culture we seem to find them chiefly comprised by the industry and sobriety of the male members of the community. It is impossible to disguise the unpleasant fact that our lower working-class mothers are ceasing to suckle their infants, not from lack of capacity to do so, nor from want of maternal instinct, but because they are too often the bread-winners of the family at a period when their whole attention ought to be devoted to their own and their infants' health. That they should be driven to this extremity is capable of several interpretations. The husbands in the class to which we allude are, for the most part, casual labourers, often with a high sense of the dignity of the male and a low estimate of the dignity of labour; men who would scorn to soil their hands with charing or other menial employments in the intervals of regular occupation so long as their wives are capable of relieving them of the necessity. Among the Jews, on the other hand, casual labour finds little favour. It is impossible to draw a strict comparison between the class of the British casual labourer and any community of the Jews, for the employments of the latter, badly paid though they may be, are for the most part regular and not liable to the extended intermittences which are the curse of the casual labouring population. But apart altogether from conditions of the labour market stands a fact of the highest significance, duly emphasised by the writer of the article to which we have above referred. "One fact," he says, "which must never be lost sight of in considering all the influences which may affect the hygienic condition of the Jew is his habitual temperance. It is not here meant to discuss the question whether the abuse of alcohol can produce a predisposition to phthisis, although many observers have thought that it may. Among the poor there is another way in which the habitual drinker menaces the health of himself and of his family, and this perhaps is more far-reaching and destructive than any direct effect which alcohol may have on the system. The cost of intoxicating liquor is a grave burden to limited earnings, and while the injurious effects of the abuse of alcohol are constantly being pointed out, it is too often forgotten that among the poor every penny spent on alcohol means a penny less on food. Thus the poor Jew, who is always temperate, has usually more to spend on food than a less temperate neighbour of equal poverty—no mean advantage in the struggle for life." If this matter of temperance be not the determining factor in the relative immunity of Jews to the tubercle bacillus, it is at least a highly-important one, and this both directly and, as we have seen, indirectly. As regards tuberculosis, it is well known that wisdom in dietary is of the utmost value no less in the matter of prevention than in that of cure. Happily the virtue of sobriety, which has already traversed the upper and middle classes with such good results, is percolating to the lower strata of society, and all the influences of the time are more favourable than they have ever been to the diffusion of knowledge upon the elementary problems of health. We may admit that the Jews are naturally endowed with a greater resistance to noxious environments than we, but after contemplating their hygienic wisdom we are guilty of cowardice if we continue in our afflictions without having made a trial of those practices of moderation and good sense in living which are so prominent among the characteristics of this remarkable people.

